# Recognizing and counting *Dendrocephalus brasiliensis* (Crustacea: Anostraca) cysts using deep learning

**DOI:** 10.1371/journal.pone.0248574

**Published:** 2021-03-18

**Authors:** Angelica Christina Melo Nunes Astolfi, Gilberto Astolfi, Maria Gabriela Alves Ferreira, Thaynara D’avalo Centurião, Leyzinara Zenteno Clemente, Bruno Leonardo Marques Castro de Oliveira, João Vitor de Andrade Porto, Kennedy Francis Roche, Edson Takashi Matsubara, Hemerson Pistori, Mayara Pereira Soares, William Marcos da Silva

**Affiliations:** 1 Faculty of Engineering, Architecture and Urbanism, and Geography, Federal University of Mato Grosso do Sul, Campo Grande, MS, Brazil; 2 College of Computing, Federal University of Mato Grosso do Sul, Campo Grande, MS, Brazil; 3 Federal Institute of Education, Science and Technology of Mato Grosso do Sul, Campo Grande, MS, Brazil; 4 Universidade Católica Dom Bosco, Campo Grande, MS, Brazil; Taipei Medical University, TAIWAN

## Abstract

The *Dendrocephalus brasiliensis*, a native species from South America, is a freshwater crustacean well explored in conservational and productive activities. Its main characteristics are its rusticity and resistance cysts production, in which the hatching requires a period of dehydration. Independent of the species utilization nature, it is essential to manipulate its cysts, such as the counting using microscopes. Manually counting is a difficult task, prone to errors, and that also very time-consuming. In this paper, we propose an automatized approach for the detection and counting of *Dendrocephalus brasiliensis* cysts from images captured by a digital microscope. For this purpose, we built the DBrasiliensis dataset, a repository with 246 images containing 5141 cysts of *Dendrocephalus brasiliensis*. Then, we trained two state-of-the-art object detection methods, YOLOv3 (You Only Look Once) and Faster R-CNN (Region-based Convolutional Neural Networks), on DBrasiliensis dataset in order to compare them under both cyst detection and counting tasks. Experiments showed evidence that YOLOv3 is superior to Faster R-CNN, achieving an accuracy rate of 83,74%, R^2^ of 0.88, RMSE (Root Mean Square Error) of 3.49, and MAE (Mean Absolute Error) of 2.24 on cyst detection and counting. Moreover, we showed that is possible to infer the number of cysts of a substrate, with known weight, by performing the automated counting of some of its samples. In conclusion, the proposed approach using YOLOv3 is adequate to detect and count *Dendrocephalus brasiliensis* cysts. The DBrasiliensis dataset can be accessed at: https://doi.org/10.6084/m9.figshare.13073240.

## Introduction

The practice of moving species is common to humans since the agriculture and cattle raising was originated, becoming more intense with the trade expansion across different parts of the world [1]. Once carried, intentionally or not by humans, those species that overcame biogeographic barriers, which another way would not allow their natural dispersal, are defined as alien species [2]. From those species, the ones that possess competitive advantages over the native can be the cause of ecological disruption by reducing biodiversity, causing the extinction of native species, being vectors, or spreading diseases [3].

Considering the presented scenario, the choice of native species for conservational efforts or productive activities must prevail when possible. Some species present characteristics that made its use viable in several areas, as such *Dendrocephalus brasiliensis*, which can be used in conservational efforts as a test organism in toxicity [4], in the residuary water treatment [5], or in productive activity of aquaculture [6]. The utilization of native species, such as, *Dendrocephalus brasiliensis*, avoids the invasive ones that can cause alterations in the environment; but for the successful adoption of a species, independent of its use, the development of technologies aiming to facilitate its management is necessary.

The main characteristics of *Dendrocephalus brasiliensis* species are the rusticity and resistance cysts production that require a period of dehydration to hatch, independent of the conditions. Thus, it is required to manipulate the cysts independent of the species utilization nature. One of the barriers to using the *Dendrocephalus brasiliensis* is the low hatching rate that, according to [7], in natural circumstances is around 7%. That way, it is necessary to work with known amounts of cysts both for experiments or commercialization aiming to achieve better hatching results.

According to [7], from 2g of cysts it is possible to produce in 2.000 *m*^2^, 2.075 g/ha/year of cysts and 1g of cyst can generate 380.000 nauplii of *Dendrocephalus brasiliensis*. However, we must consider that the number of cysts present in the substrate may vary according to matrices quality, medium conditions, or any stress that can disrupt the capacity or amount of laying. Besides, working with clean cysts without the substrate is a very laborious task that involves the use of several meshes to help separate cyst and dirt, which is also very time-consuming [8]. Due to the low hatching rate of the *Dendrocephalus brasiliensis* species, by adopting a gram of the substrate without separating the cysts can create results that are not befitting with reality when used in an experiment. For example, if 1g of substrate that contains almost no cysts is adopted, with the low hatching rate, the results are likely to be different from reality because of data inaccuracy.

There are two options to ensure data accuracy: the manual counting of the cysts present in the substrate or; perform the cleaning and separation of the cysts from the dirt. However, both tasks are very laborious and time-consuming. In this way, alternatives must be developed to improve accuracy and facilitate the cysts counting process. An alternative is to automate the process using domain-specific object detection techniques based on computer vision. These techniques deal with detecting instances of objects of a determined category (such as fruits, fish species, or cysts) in digital images based on features like shape, color, texture, etc [9].

In recent years, several architectures based on computer vision for object detection have emerged and have made significant advances. Among these architectures, YOLO (You Only Look Once) [10] and Faster R-CNN (Region-based Convolutional Neural Networks) [11] and their variants stand out due to a wide range in real-world applications. Some researchers have used object-detection based approaches in productive activities such as precision agriculture and also in conservation of species. In the conservation efforts, [12] used a unified approach based on YOLO to detect and classify fish in underwater videos, whose fish species classification accuracy varied from 79.8% to 91.64%. [13] also achieved satisfactory results by adopting YOLO to detect and track fish underwater, however, they used images captured from web cameras placed above the pond instead of underwater videos. In the context of precision agriculture, [14] used several detection methods, including Faster R-CNN and YOLOv3, to detect pests that dominantly attack field crops in order to real-time monitor them. YOLOv3 and Faster R-CNN obtained an average precision in detection of pests of 63.54% and 51.72%, respectively. [15] achieved video-based fruit counting performances up to 93% on three different fruits using Faster R-CNN. [16] presented an architecture with two-stage to detect aphid, whose detection stage is based on YOLO. The experiments showed that the approach achieved an aphid detection performance of 76.8% average precision. [17] used an approach based on Faster R-CNN to obtain images from maize seedlings to distinguish maize seedlings and weeds in crops. The approach obtained an average precision in the detection of maize seedlings with respect to soil and weeds of 97.71%. [18] used Faster R-CNN to detect and count banana plants on a farm using aerial images collected from a UAV (Unmanned Aerial Vehicle). The approach achieved 97.9%, 91.5%, and 87.2% accuracy on altitudes of 40m, 50m, and 60m, respectively. These are some examples of process automation using domain-specific object detection techniques based on computer vision.

In this paper, we compare state-of-the-art object detection models Faster R-CNN and YOLOv3 in order to propose an automated approach for *Dendrocephalus brasiliensis* cysts detection and counting from images obtained by a digital microscope. Besides, we show that it is possible to infer the number of cysts from a substrate with a known weight. Finally, we introduce the DBrasiliensis dataset, a repository with 246 images containing 5141 cysts of *Dendrocephalus brasiliensis*, a native species from South America. One of the motivations for publishing the DBrasiliensis dataset is related to the importance and potential of this species to productive activities in aquaculture and conservational efforts. A dataset with cysts examples can help to accelerate researches that need *Dendrocephalus brasiliensis* cysts in an automated way using computer vision, as well as new applications for counting and weight inference of cysts.

The contributions of this paper are:

- The publication of a novel annotated *Dendrocephalus brasiliensis* cysts images dataset, called DBrasiliensis, composed of 246 images divided into training and testing. The training set has 111 images containing 3173 annotated cysts. The testing set has 135 images divided into ten subsets, whose labels represent the weight in grams of each one. In all, the testing set has 1968 cyst images. To the best of our knowledge, this is the first *Dendrocephalus brasiliensis* cysts image dataset destined for deep learning. The DBrasiliensis dataset can be accessed at: https://doi.org/10.6084/m9.figshare.13073240.
- Definition of a baseline for detection and counting *Dendrocephalus brasiliensis* cysts using the state-of-the-art YOLOv3 and Faster R-CNN.
- A deep learning-based automatized approach to detect and count *Dendrocephalus Brasiliensis* cysts from images obtained by a digital microscope.

The rest of the paper is organized as follows. In the Materials and Methods Section, we describe the DBrasiliensis dataset, introduce an overview of the YOLOv3 and Faster R-CNN, and also present the experimental setup, followed by the analysis of results in the Results and Discussion Section and conclusions in Conclusion Section.

## Materials and methods

### DBrasiliensis dataset

**Fig 1:**
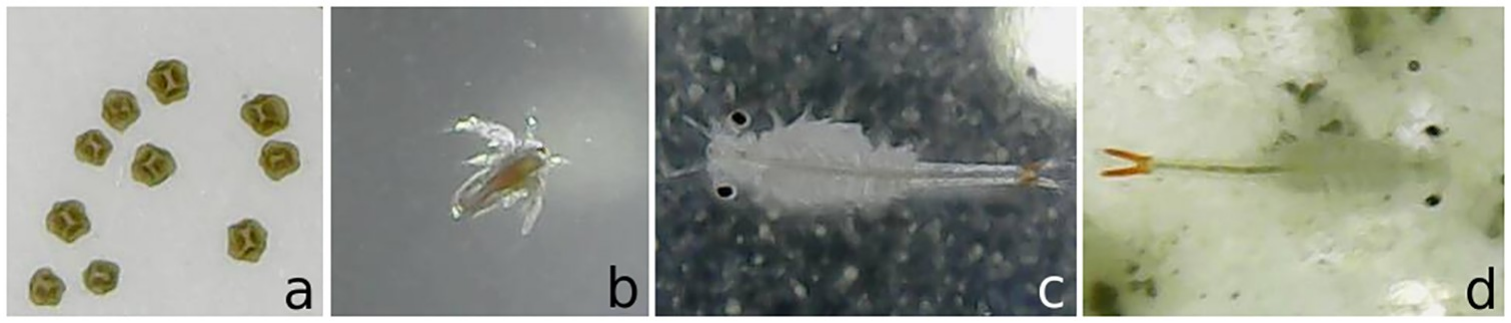
Life stages of *Dendrocephalus brasiliensis*: a) cysts, b) nauplius, c) juvenile, and d) adult.

**Fig 2:**
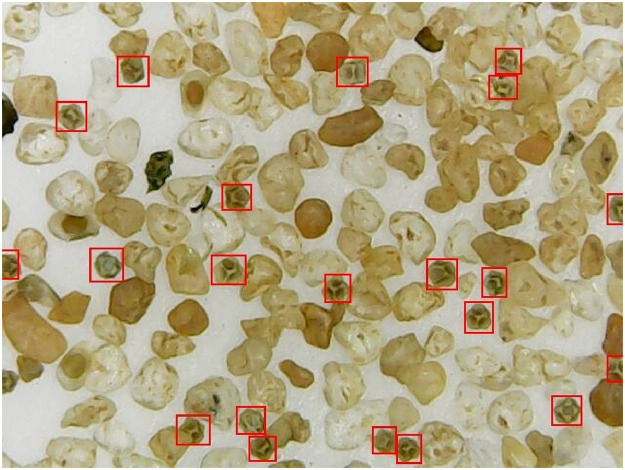
Substrate image captured by the XTRAD USB digital microscope model XT-2036 at a 52x magnification. Each red rectangular bounding box displays a cyst.

The *Dendrocephalus brasiliensis*, whose life stages is presented in Fig 1, lays resistance cysts in the bottom of culture medium, such as an aquarium, small lakes, etc. These cysts mix with the substrate present at the bottom of the culture medium which is basically composed of organic and inorganic matter. Fig 2 shows a substrate sample, whose cysts are highlighted by a red rectangular bounding box.

In order to build the DBrasiliensis dataset, we took substrates portions from the bottom of an aquarium that we used as an incubator for the *Dendrocephalus brasiliensis* and split them into two parts: one to capture the training images and the other to capture the test images. Both parts were fixed on white coverslips in order to be observed using a digital microscope. We used an XTRAD USB digital microscope model XT-2036 with 52x magnification to capture the images with resolution of 640 × 480 pixels.

**Fig 3:**
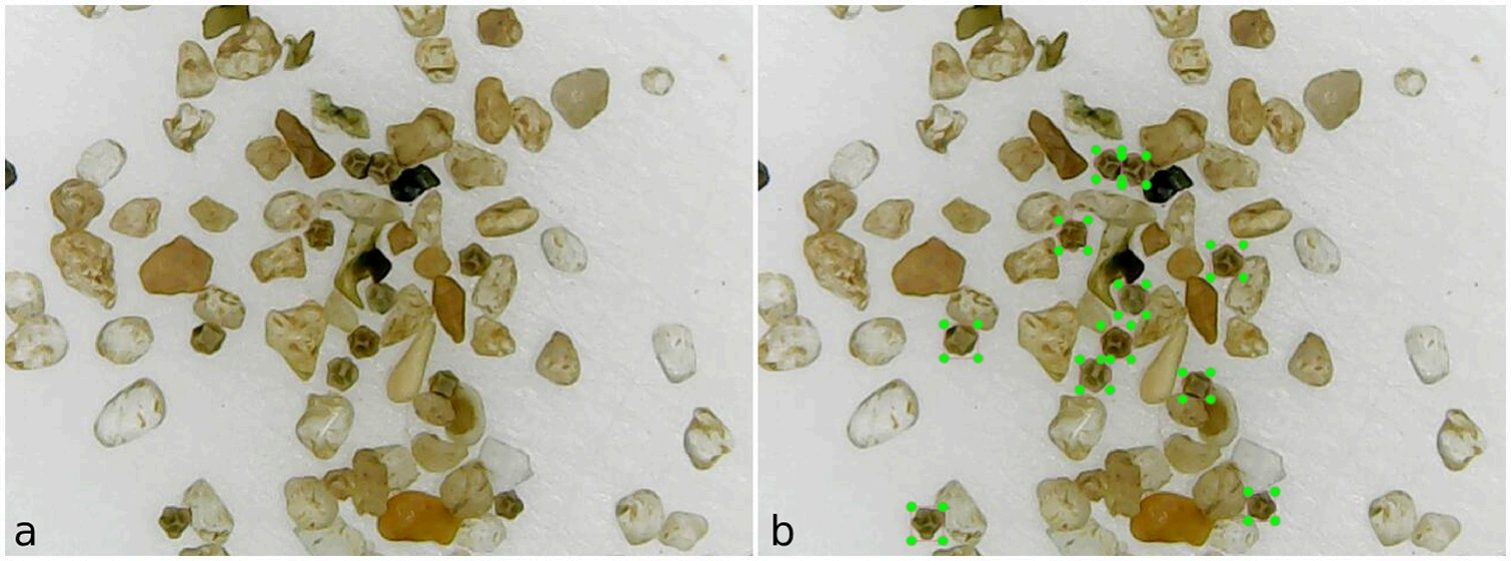
The labeling process using the LabelImg software: a) Sample of substrate image captured by the XTRAD USB digital microscope model XT-2036 at a 52x magnification; b) Using the LabelImg software to label samples of cysts. The green rectangles in the image are the labeled cysts.

On the images designated to the training set (see example in Fig 3(a)), we used the LabelImg software to label cysts in both PASCAL VOC [19] and YOLO formats, as shown in Fig 3(b). There are 111 images in all, for training, containing 3173 annotated cysts.

**Fig 4:**
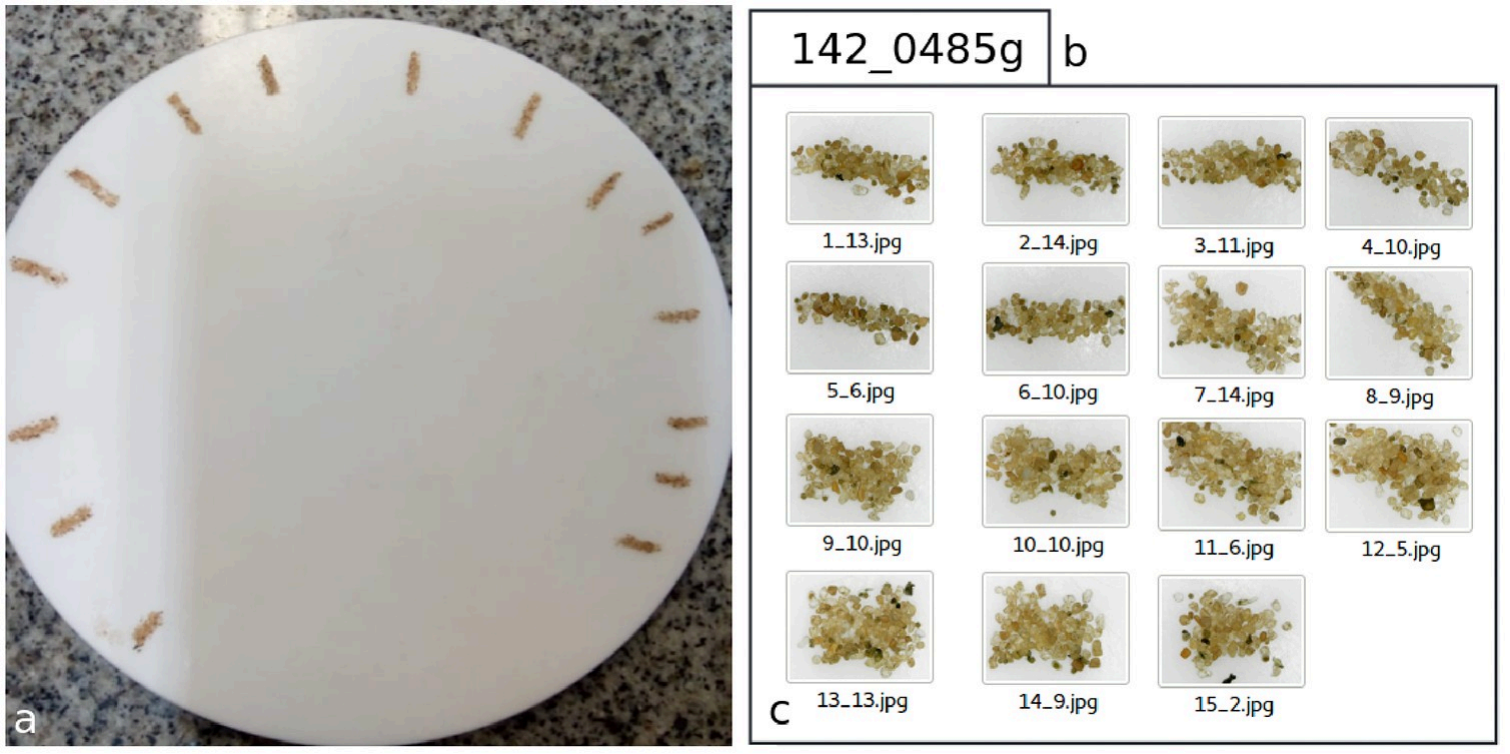
The testing set building process: a) The substrate split into small portions on a white coverslip. b) Folder name (label) that indicates the amount of cyst (142) and the weight of the substrate (0.485g) contained in the folder. c) Sample of images in folder captured by the XTRAD USB digital microscope model XT-2036. The file name indicates the image number and amount of cyst contained in it. For example, in the 1_13.jpg file, 1 indicates the file number and 13 indicates the number of cysts in the image.

We divided the images designated for the tests into ten small groups. Each small group received a label that indicates the number of cysts in the images group and the weight of the substrate used to capture the images. For building a given group, we split the substrate reserved to it into small portions on a white coverslip and weighed it using a precision scale (see Fig 4(a)). Then, we captured an image of each portion of the substrate using the digital microscope. The captured images were stored in a folder, whose name (label) indicates the amount of cyst and the substrate weight contained in the image group (see Fig 4(b)). Besides, the file name of each image in the folder indicates the image number and amount of cyst contained in it (see Fig 4(c)). The complete testing set has 1968 cysts in 135 images distributed in 10 folders (10 small image groups). The substrate weight used to build the testing set is 4.24 grams. Table 1 shows the testing set in detail.

**Table 1:** The testing set: 10 folders, 135 images, 4.24 grams of substrate, and 1968 cysts.

Folder (label)	Weight (grams)	Number of images	Number of cysts
70_0407g	0.407	14	70
142_0485g	0.485	15	142
149_0420g	0.420	13	149
165_0559g	0.559	16	165
196_0459g	0.459	14	196
213_0223g	0.223	11	213
219_0333g	0.333	14	219
239_0479g	0.479	12	239
256_0419g	0.419	12	256
319_0456g	0.456	14	319

### An overview of Faster R-CNN and YOLOv3 architectures

YOLOv3 [10] and Faster R-CNN [11] are state-of-the-art object detection architecture and are employed to solve many problems whose aim is to detect and classify objects [9]. In this section, we provide an overview of both the architectures.

#### YOLOv3 architecture

The YOLOv3 workflow is basically composed of three steps [10]. First, it receives an input image and then divides it into a grid. Next, it applies the image classification and localization processes on each grid cell in order to predict class probabilities for objects and their corresponding bounding boxes. For both classification and localization processes, the YOLOv3 uses an open-source CNN (Convolutional Neural Network) called Darknet-53 as backbone, whose 53 first layers are for classification and another 53 additional layers are for detection, resulting in a CNN with a total of 106 layers.

The object detection is done at three different scales in 82nd, 94th, and 106th layers, whose inputs are downsampled by a factor of 32, 16, and 8, respectively. The 82nd layer is responsible for detecting large objects, the 94th layer for medium objects, and the 106th layer for smaller objects. The detection at different layers provides detection of small objects since the upsampled layers are concatenated with the previous layers in order to preserve the object’s fine-grained features. During the detection multiple bounding boxes for each object in a grid cell can be predicted. To define the right bounding box for the object, the IoU (Intersection over Union) is calculated between bounding boxes in the grid cell and is selected one with the highest IoU. For those bounding boxes selected, the network calculates conditional class probabilities. Finally, conditional class probabilities and box confidence predictions jointly provide class-specific confidence scores for each bounding box [10].

The Darknet-53 architecture, used by YOLOv3 as a backbone, is mainly composed of successive 3 × 3 and 1 × 1 convolutional layers. Each convolution layer is followed by a Batch Normalization layer [20] and Dropout operations [21]. At the end of each convolutional block, residual blocks are added in order to perform the identity mapping, whose purpose is to add the output from the previous convolutional layer *x* to output *F(x)* of the layer ahead. This allows *x* and *F(x)* to be combined as input to the next convolutional layer [22]. The final block consists of a Global Average Pooling [23] followed by a fully connected layer and a final layer Softmax [24].

**Fig 5:**
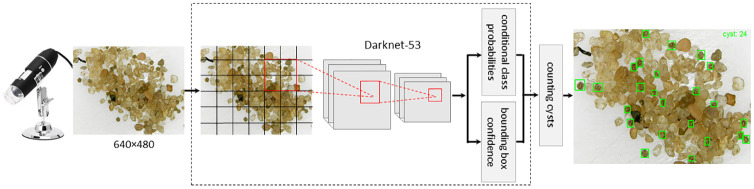
Overview of the automatized approach for *Dendrocephalus Brasiliensis* cysts detection and counting using YOLOv3.

Fig 5 shows the general workflow of YOLOv3 applied to cyst detection and counting. After the YOLOv3 was trained using the annotated images of the DBrasiliensis dataset designated for training, a test image captured by the digital microscope is inputted into the model to detect the cysts. Next, the image is divided into several grid cells. For each cell there are predicted several anchor boxes and confidence scores. Then, the boxes with the highest score are selected so that the network calculates conditional class probabilities for each one. For the last step, the conditional class probabilities and box confidence predictions jointly provide cyst class confidence scores for each box, drawing a bounding box around each cyst in the image. We design a post-processing step that counts the cysts detected in the image.

#### Faster R-CNN architecture

The Faster R-CNN is composed of two modules [11]: RPN (Region Proposal Network) and Fast R-CNN detector. The RPN receives as input an image that is processed by a CNN in order to obtain features and produce a set of rectangular region proposals with three scales (128 × 128, 256 × 256 and 512 × 512) and three aspect ratios (1:1, 2:1 and 1:2) that possibly have the candidate objects. The Fast R-CNN detector receives input RoIs (Region of Interest) produced from the region proposals generated by RPN. Each RoI is processed by a pooling layer and pooled into a fixed-size feature map that is mapped to a feature vector. This feature vector will be the input for a fully connected layer to classify the RoI. The output is composed of two vectors per RoI: the probabilities and bounding-box for each object class considered. Both RPN and Fast R-CNN detector modules share a common set of convolutional layers which can be provided by a CNN backbone like VGG16 [25], ResNet-50 [22], or Inception-v2 [26]. In this paper, we choose the Inception-v2 architecture to act as a backbone for Fast R-CNN.

Inception-v2 architecture [26] has three initial convolutional layers with 3 × 3 filters followed by max-pooling. The output of this block is the input for another block with three convolutional layers with 3 × 3 filters. Next, the architecture has three inception modules in sequence. In the first module, it is performed convolution on an input using filters 1 × 1 and 3 × 3, as well as max-pooling. The resulting outputs are concatenated and moved to the next inception module that applies a grid reduction technique to reduce the number of parameters in order to become the model computationally cheaper. The grid reduction consists of 1 × n and n × 1 convolutions instead of n × n convolutions. Like in the first inception module, the outputs are concatenated and moved to the next inception module. The last inception module is similar to the second, however, it is wider instead of deeper. Finally, before the final layer Softmax, an extra classifier act as a regularizer [26].

**Fig 6:**
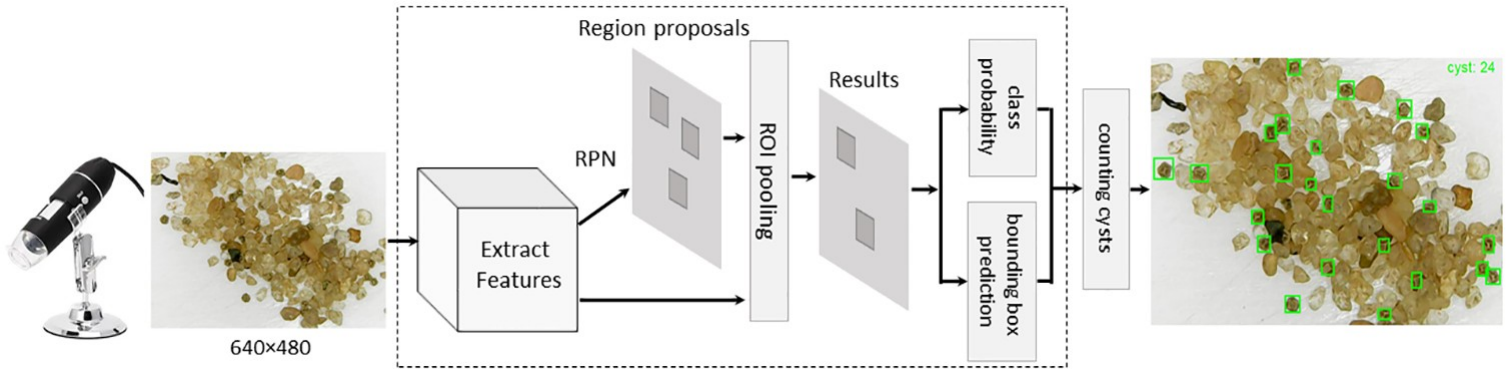
Overview of the automatized approach for *Dendrocephalus Brasiliensis* cysts detection and counting using Faster R-CNN architecture.

Fig 6 shows the general workflow of Faster R-CNN applied to cyst detection and counting. After the training, a test image captured by the digital microscope is inputted into the model to detect the cysts. The image passes through convolutional layers to obtain feature maps, which are inputted into RPN to generate rectangular region proposals. The region proposals are transformed into RoIs and inserted into the Fast R-CNN process that provides cyst class probability and bounding box prediction for each one. Finally, a post-processing step counts the cysts detected in the image.

### Experimental setup

Both YOLOv3 and Faster R-CNN architectures were set to use the fine-tuning strategy with all layers initialized with weights from previous training on the MS-COCO (Microsoft Common Objects in COntext) dataset [27]. Besides, we set the learning rate at 0.001, the number of iterations at 8,000, and varied the batch size at 2, 4, 8, 16, 32, and 64. We used a small batch size to consume less memory and train the architectures faster since the small batch size allows us to update the network weights more often [28]. We limited the number of iterations to 8,000, as from that number, the loss rate did not present improvement. We set the learning rate at 0.001 because this value is recommended by [29] when used a small number of samples on training. During training, all images in the batch were augmented using random rotation by +30/-30° and exposure between -10% and +10%. Both architectures were trained using a Tesla P100-PCIE-16GB GPU.

We used the DBrasiliensis dataset to train and test both architectures. Thus, 111 images containing 3173 annotated cysts were used in training, i.e., 61.72% of the cysts, and in the test were used 135 images with 1968 cysts, 38.28% of the cysts, arranged into 10 different subsets as presented in Table 1. Each built model has been tested ten times using only one test subset at a time. The metric result is an average from the sum of the scores achieved on each one test subset.

We considered a correct detection (true positive) when the predicted cysts have a detection score of ≥ 0.3, and a wrong detection (false positive) when the detected object isn’t a cyst. A false negative is assigned when a cyst is in the image and it isn’t detected. The evaluation metrics used were Precision, F1-Score, Accuracy, and Recall. In all formulas below, TP refers to true positives, TN to true negatives, FN to false negatives, and FP to false positives.

$$\begin{array}{c} \text{Accuracy}=\frac{\text{TP}+\text{TN}}{\text{TP}+\text{FP}+\text{FN}+\text{TN}} \end{array}$$ (1)

$$\begin{array}{c} \text{Precision}=\frac{\text{TP}}{\text{TP}+\text{FP}} \end{array}$$ (2)

$$\begin{array}{c} \text{Recall}=\frac{\text{TP}}{\text{TP}+\text{FN}} \end{array}$$ (3)

$$\begin{array}{c} \text{F}1-\text{Score}=\frac{2*(\text{Recall}*\text{Precision})}{\left(\text{Recall}+\text{Precision}\right)} \end{array}$$ (4)

Besides, we evaluated both architectures in terms of MAE (Mean Absolute Error), RMSE (Root Mean Square Error), and R^2^. Finally, we applied statistical methods on the Accuracy metric to evaluate the differences among the architectures.

We performed a statistical analysis using the Shapiro-Wilk test [30] to verify the normality of the data, the one-way Anova hypothesis test, and the Tukey’s test [31] to analyze the difference between the architectures in a pairwise way. We adopted a significance level of 5% for all statistical tests (p-value < .05).

## Results and discussion

The classification results for Precision, F1-Score, Accuracy, and Recall for both architectures are presented in Table 2. Table 2 shows that the YOLOv3 architecture exhibits higher and more uniform precisions than the Faster R-CNN, indicating that the proportion of true positives concerning the total of predicted positives achieved by it didn’t present large distortions. It is important to emphasize that the YOLOv3 achieved the best results with batch size set at 32, except the precision, whose best index was achieved with the batch size set at 4.

**Table 2:** Cyst detection results at average percentage through 10 test subsets on the DBrasiliensis dataset for YOLOv3 and Faster R-CNN.

Architecture	batch size	Precision	Recall	F1-Score	Accuracy
YOLOv3	2	97.63 ±2.86	77.28 ±5.18	86.11 ±3.06	75.76 ±4.80
	4	**99.54** ±0.76	67.91 ±4.68	80.65 ±3.28	67.69 ±4.56
	8	99.19 ±0.49	68.59 ±7.29	80.89 ±5.08	68.20 ±7.20
	16	99.34 ±0.91	70.39 ±2.55	82.38 ±1.63	70.06 ±2.32
	32	98.24 ±1.31	**85.05** ±5.01	**91.05** ±2.81	**83.73** ±4.76
	64	99.07 ±1.12	84.07 ±3.88	90.89 ±2.23	83.40 ±3.78
Faster R-CNN	2	79.72 ±4.20	**59.66** ±8.24	**67.86** ±5.62	**51.65** ±6.33
	4	**94.44** ±3.64	40.72 ±4.84	56.77 ±5.11	39.82 ±4.95
	8	87.25 ±3.36	47.07 ±5.63	60.93 ±4.86	43.99 ±5.00
	16	88.22 ±5.40	37.86 ±5.47	52.81 ±5.83	36.10 ±5.43
	32	93.88 ±2.03	35.89 ±4.88	51.73 ±5.08	35.05 ±4.65
	64	92.95 ±1.65	35.02 ±4.47	50.70 ±4.67	34.09 ±4.22

Bold font indicates the best results obtained by each architecture.

**Fig 7:**
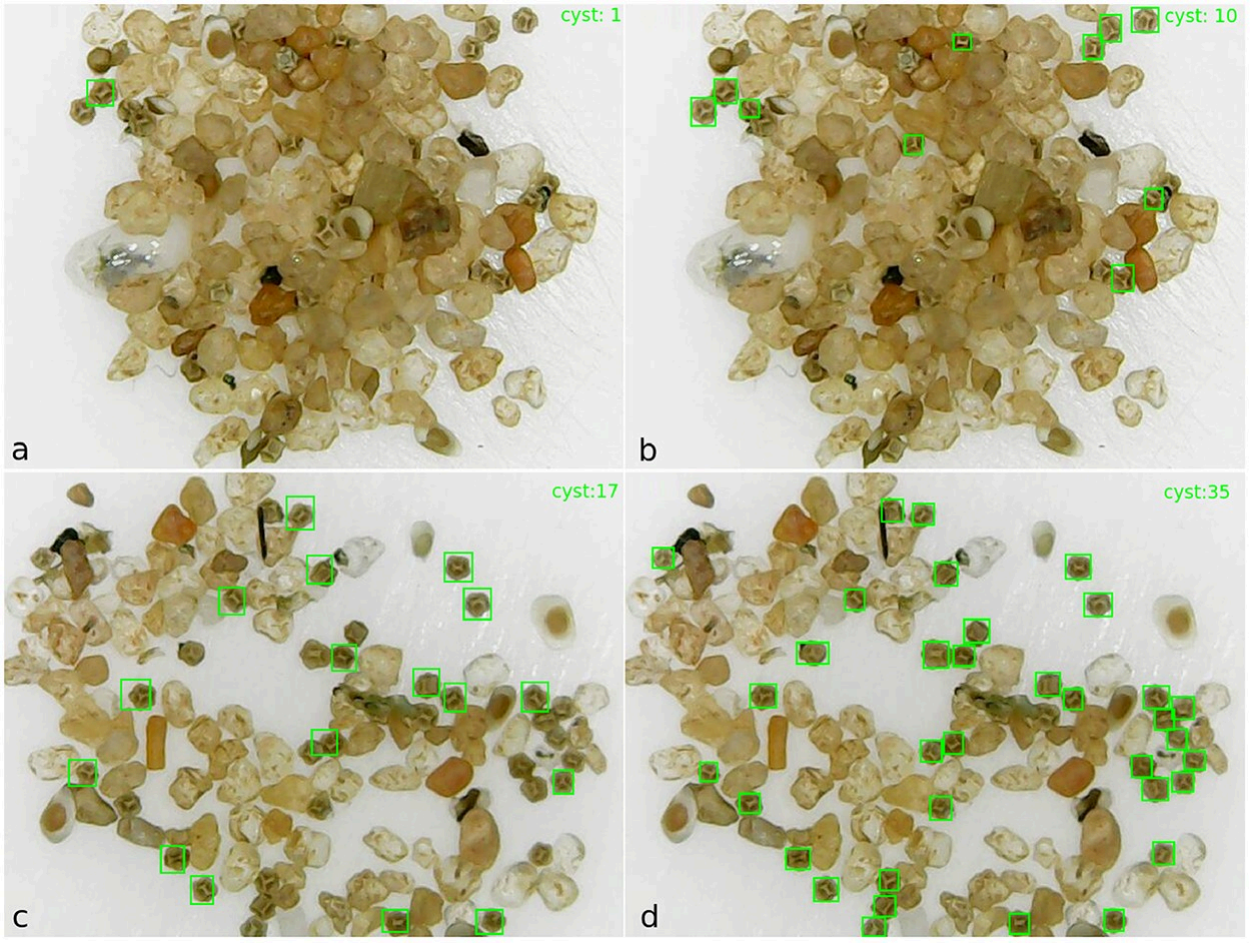
Example of detecting and counting cysts of the Faste R-CNN and YOLOv3 architectures: a) The Faster R-CNN detected and counted only 1 of 20 cysts in the image; b) The YOLOv3 detected and counted 10 of 20 cysts in the image; c) The Faster R-CNN detected and counted 17 of 36 cysts in the image; d) The YOLOv3 detected and counted 35 of 36 cysts in the image.

The Faster R-CNN achieved 94.44% of precision with batch size set at 4, showing that the observed true positives really were cysts. However, it presented a high false negatives rate that can be observed at the recall of 40.72%. One example of this high false negative rate can be seen in Fig 7(a). From the 20 cysts in the image, the Faster R-CNN detected only 1. On the other hand, the YOLOv3 with batch size also set at 4, in which it achieved the best precision and recall of 67.91%, detected 10 of 20 cysts in the same image (see Fig 7(b)).

**Fig 8:**
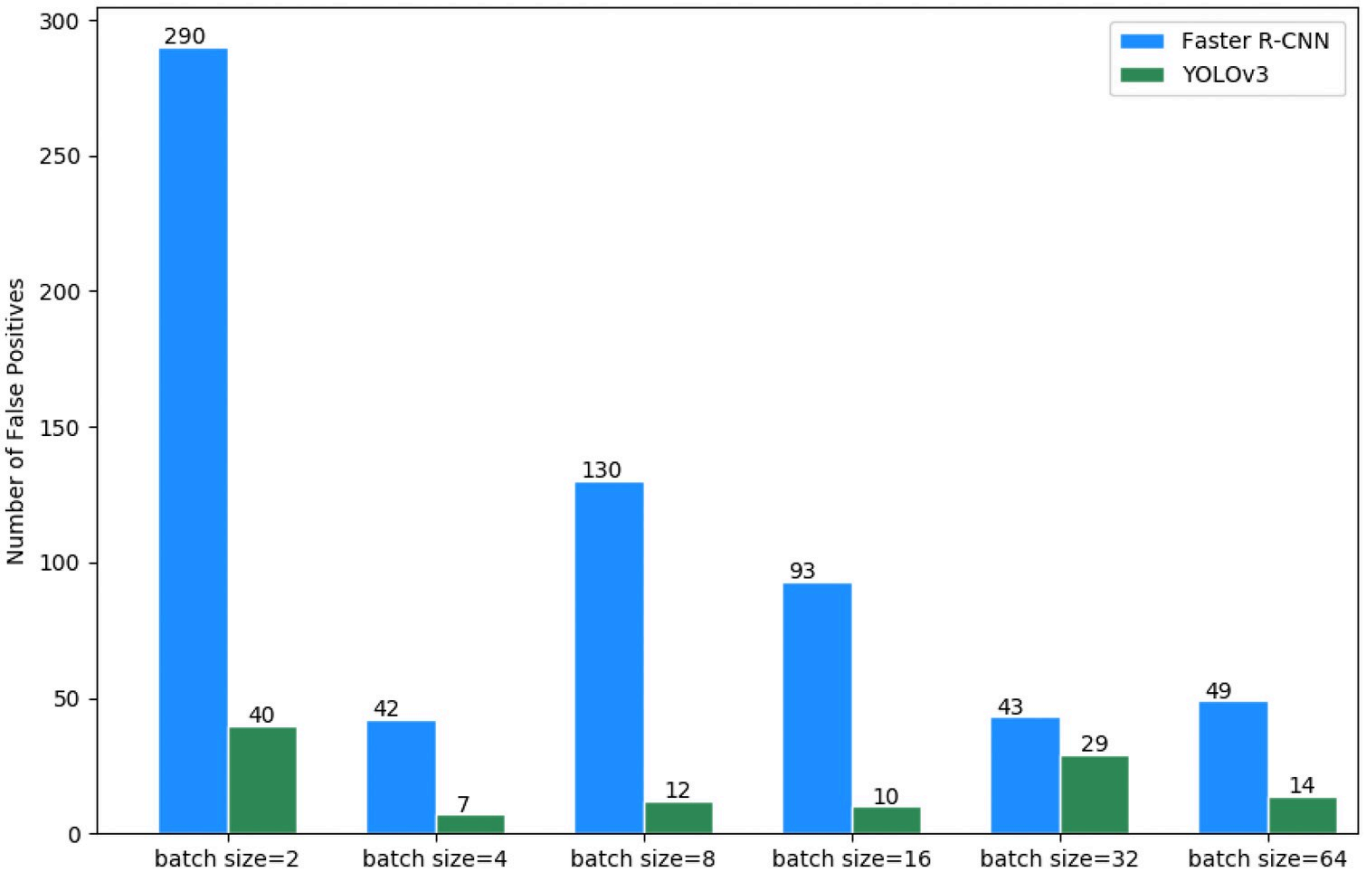
Comparison of the number of false positives of the Faster R-CNN and YOLOv3 architectures in different batch sizes.

We can observe in Table 2 that both YOLOv3 and Faster R-CNN with batch size set at 4 achieved better precision. Nevertheless, for other metrics, the YOLOv3 and Faster R-CNN achieved better results with batch size set at 32 and 2, respectively. This high precision with batch size at 4 relates to the low false positive rates achieved by the architectures. YOLOv3 with batch size set at 4 got better precision because it had false positive rates lower than when batch size is 32. Notice in Fig 8 that YOLOv3 with batch size set at 4 got only 7 false positives. On the other hand, when the batch size is 32, the number of false positives is 29. The same is true of the Faster R-CNN with batch sizes set at 2 and 4, whose number of false positives were 290 and 42, respectively (see Fig 8). However, the high precision of the architectures with batch size at 4 did not translate into detecting more cysts (see Table 3).

**Table 3:** Detection and counting results for all batch sizes on the 10 testing subsets of the DBrasiliensis dataset for YOLOv3 and Faster R-CNN.

	Yolov3						Faster-RCNN					
	Hits in each test set (folder) per batch size						Hits in each test set (folder) per batch size					
cyst in set	2	4	8	16	32	64	2	4	8	16	32	64
70	50	49	45	49	60	59	49	27	32	22	27	26
142	117	98	107	102	123	123	90	59	71	55	53	52
149	114	99	107	104	123	117	107	71	83	67	60	58
165	119	114	103	115	134	135	102	74	74	67	64	62
196	156	127	132	140	174	167	130	91	106	94	88	85
213	169	146	147	146	188	182	121	71	110	76	74	73
219	180	163	163	164	194	190	128	93	99	79	75	73
239	206	180	197	174	224	220	129	102	112	96	78	77
256	181	153	143	166	196	204	118	91	96	81	71	70
319	231	197	201	224	250	256	153	109	125	100	95	94
Total:1968	1523	1326	1345	1384	**1666**	1653	**1127**	788	908	737	685	670

Bold font indicates the best results obtained by each architecture.

Concerning accuracy, the Faster R-CNN achieved the lowest results at all batch sizes compared to YOLOv3. The best accuracy rate achieved by YOLOv3, 83.73%, is relevant due to the difficulty of detecting cysts in the substrate because, in many instances, only parts of the cyst are visible, or the cysts are glued together, and there is also a considerable quantity of sand and other residues. Fig 7(c) and 7(d) show examples of detection of the Faster R-CNN and YOLOv3, respectively, using the same image and batch size set at 2 for Faster R-CNN and 32 for YOLOv3. Although there are no false positives in both images, the Faster R-CNN presented 19 false negatives, by detecting 17 of 36 cysts. On the other hand, the YOLOv3 presented only 1 false negative, i.e., it detected 35 of 36 cysts in image. This difference in accuracy between the architectures was repeated in most of the images of the DBrasiliensis dataset reserved for testing.

We counted the number of false positive detections in different batch sizes (see Fig 8). This count confirms the precision achieved by each architecture configurations shown in Table 2, i.e., the precision decrease as the number of false positive increases.

**Fig 9:**
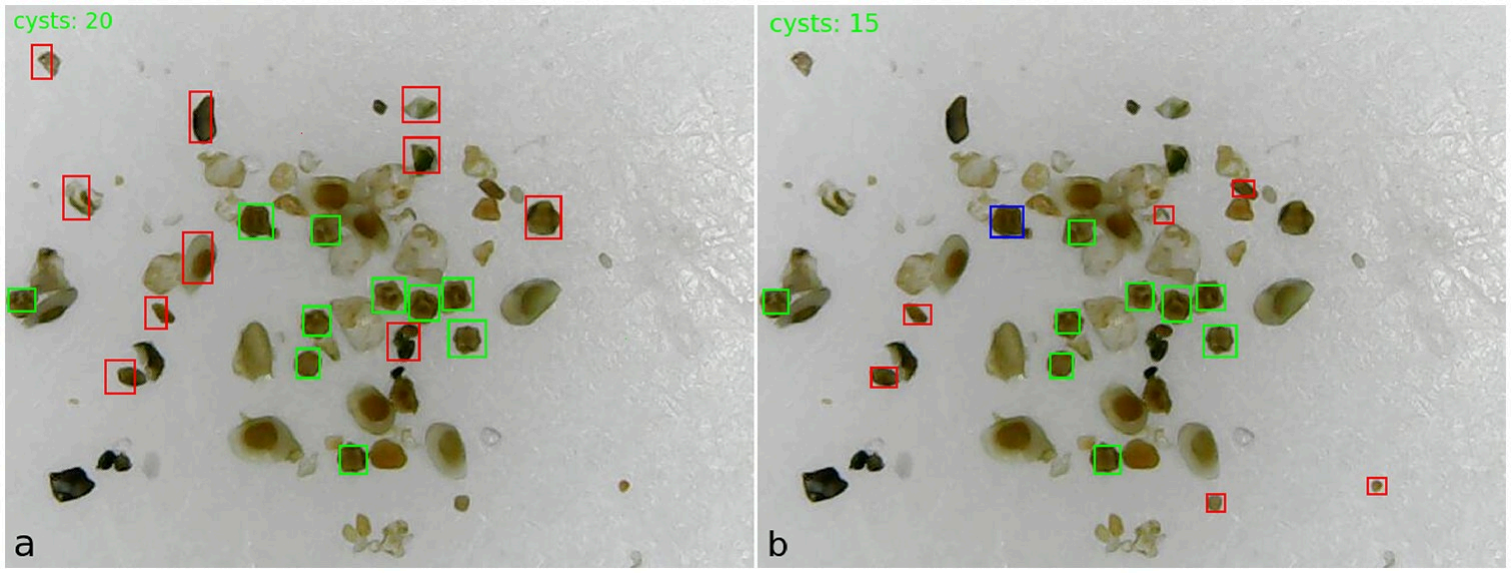
Example of detecting and counting cysts of the Faster R-CNN (a) and YOLOv3 (b) architectures, both with batch size set at 2. Green boxes are true positives with score detection ≥ 0.3, red boxes are false positives, and blue boxes are false negatives.

We analyzed the false positives and observed that most of them have similar colors to cyst colors and, in some cases, they have parts similar to cyst shapes. Fig 9(a) and 9(b) show an image in which both architectures, Faster R-CNN and YOLOv3, with batch size set at 2, achieved a high false positives rate. The red rectangular bounding boxes show the false positives, the greens the true positives, and the blues the false negatives.

Table 3 shows the detection and counting results for all batch sizes on the 10 testing subsets. It can be noted that YOLOv3 outperforms the Faster R-CNN on all testing subsets and has a higher hit rate with batch size set at 32, detecting and counting 1666 of 1968 cysts, a hit percentage of 84.66%. The Faster R-CNN achieved the higher hit percentage with batch size set at 2, detecting and counting 1127 of 1968 cysts, 57.27%.

We carry out an analysis of variance with Anova on a .05 level of significance using the accuracy as metric to determine if there is a difference between the different batch sizes in each architecture, as well as if there is some difference between the average accuracy of both architectures. We adopted the Anova test because, in general, the average accuracy presented normality/homogeneity of variance after we performed the test of normality using the Shapiro-Wilk.

The test between the different batch sizes for Faster R-CNN resulted in a p-value of 0.0001, which indicates a statistically significant difference between the different batch sizes average accuracy. The Tukey test showed that the batch size defined in 2 differs from the others.

The test between the different batch sizes for YOLOv3 also resulted in a p-value of 0.0001, indicating a significant difference between the batch sizes average accuracy, except the batch sizes set at 32 and 64 which according to Tukey test didn’t present a statistically significant difference between them.

The comparison between YOLOv3 and Faster R-CNN using the accuracy as metric resulted in a p-value < .05, indicating a statistically significant difference between the models.

Table 4 shows that YOLOv3 reached R^2^ of 0.88 for batch sizes 32 and 64. On the other hand, Faster R-CNN achieved the best R^2^ using batch size 2 (0.20). These results indicate that YOLOv3 outperforms Faster R-CNN in detecting and counting the cysts since the R^2^ metric is a performance indicator, and the higher the result the better the agreement between the resulting count of the architectures and the number of cysts in the DBrasiliensis Dataset.

**Table 4:** RSME, MAE, and R^2^ through 10 test subsets on the DBrasiliensis dataset for YOLOv3 and Faster R-CNN.

Architecture	batch size	RMSE	MAE	*R*^2^
YOLOv3	2	4.91	3.30	0.77
	4	6.49	4.76	0.59
	8	6.38	4.61	0.61
	16	6.07	4.33	0.64
	32	**3.49**	**2.24**	**0.88**
	64	3.57	2.33	**0.88**
Faster R-CNN	2	**9.13**	**6.23**	**0.20**
	4	11.64	8.74	-0.31
	8	10.58	7.85	-0.08
	16	12.03	9.12	-0.40
	32	12.39	9.50	-0.48
	64	12.50	9.61	-0.51

Bold font indicates the best results obtained by each architecture.

In terms of RSME and MAE, Table 4 shows that YOLOv3 using batch size 32 achieved 3.49 and 2.24, respectively. This result indicates that YOLOv3 has the lowest average standard deviation using batch size 32 between the number of cysts detected and counted and the number of cysts in the DBrasiliensis dataset. From this result, we can tell that YOLOv3 with batch sizes set at 32, and the learning rate at 0.001 is the approach best suited to detect and count cysts, since that configuration achieved the best results for Accuracy, Precision, R^2^, RSME e MAE.

We also carry out an analysis of variance with Anova on a.05 level of significance using the accuracy as a metric to determine if the YOLOv3 with batch size set at 32 maintains the average accuracy between the different testing subsets (see Table 1). The test resulted in a p-value of 0.1008, therefore, we have no evidence that there is a statistically significant difference in YOLOv3 accuracy on the different testing subsets of the DBrasiliensis dataset.

In that way, taking into account that the testing set of the DBrasiliensis dataset consists of 10 subsets, each of which is associated with the substrate weight used to capture your images (see Section DBrasiliensis dataset), we can infer the number of cysts for a new portion of substrate with a known weight obtained from the same aquarium where we took the substrate to build the DBrasiliensis dataset. Thus, a producer or researcher associating weights and counts can use the same technique to infer his production.

The inference of the number of cysts from the substrate with a known weight, for both research and cultivation, is a necessary practice because the manual counting of thousands of cysts is not feasible. Thus, we can use YOLOv3, with batch size set at 32, to infer the total number of cysts contained in a substrate, counting a certain number of cysts collected through sampling, with the samples vary according to the need for more/less accuracy of the data. Adopting the inference, we will be able to count cysts with 83.73% of accuracy (Table 2). It is up to the producer or scientist to analyze the number of samples (set of images associated with a weight) that best suits their needs.

Although the proposed approach can present disadvantages, such as the work required for annotation of thousands of cysts and the computational cost for training the model, the benefit obtained by it, concerning the accuracy and the counting time, is a factor that supports the adoption of automated cyst counting. For instance, the YOLOv3 takes around 1 minute and 29 seconds to count the cysts of 135 images with 83.73% of accuracy and 98.24% of precision.

We believe that the results obtained by YOLOv3 with batch size set at 32 are enough to build an automatic detection and counting system of cysts since the visual counting of cysts performed by humans using microscopes is a hard task, prone to errors, and that also very time-consuming. Besides, it is possible to optimize the process of gauging the number of cysts present in a given medium or substrate, inferring the number of cysts without the need for cleaning, drying, and manual counting.

## Conclusion

Due to the potential of *Dendrocephalus brasiliensis* species in the conservational efforts and productive activities, we presented a new technology aimed to improve and facilitate the cysts measurement process. We built a novel annotated images dataset of *Dendrocephalus Brasiliensis* cysts called DBrasiliensis and used the YOLOv3 and Faster R-CNN to provide a baseline for detecting and counting cysts. To promote research in the automation of cyst measurements, we also report evidence that the performance of YOLOv3 is superior against Faster R-CNN. Besides, we provided the possibility of inferring the total number of cysts, with an accuracy around 83.73%, from a substrate image set associated with a known weight. The DBrasiliensis dataset can be accessed at: https://doi.org/10.6084/m9.figshare.13073240.
